# Epithelial cell adhesion molecule overexpression regulates epithelial-mesenchymal transition, stemness and metastasis of nasopharyngeal carcinoma cells via the PTEN/AKT/mTOR pathway

**DOI:** 10.1038/s41419-017-0013-8

**Published:** 2018-01-05

**Authors:** Meng-He Wang, Rui Sun, Xiao-Min Zhou, Mei-Yin Zhang, Jia-Bin Lu, Yang Yang, Li-Si Zeng, Xian-Zi Yang, Lu Shi, Ruo-Wen Xiao, Hui-Yun Wang, Shi-Juan Mai

**Affiliations:** 1 State Key Laboratory of Oncology in South China, Guangzhou, China; 2 Collaborative Innovation Center for Cancer Medicine, Guangzhou, China; 3 Department of Nasopharyngeal Carcinoma, Sun Yat-Sen University Cancer Center, Guangzhou, China; 4 Zhoukou Hospital of Traditional Chinese Medicine, Zhoukou, China; 5 Department of Pathology, Sun Yat-Sen University Cancer Center, Guangzhou, China; 6 Cancer Center of Guangzhou Medical University, Guangzhou, China

## Abstract

Epithelial cell adhesion molecule (EpCAM) is known to be highly expressed in a variety of epithelial carcinomas, and it is involved in cell adhesion and proliferation. However, its expression profile and biological function in nasopharyngeal carcinoma (NPC) remains unclear. In this study, higher expression of EpCAM was found in NPC samples compared with non-cancer nasopharyngeal mucosa by qRT-PCR. Additionally, immunohistochemistry (IHC) analysis of NPC specimens from 64 cases showed that high EpCAM expression was associated with metastasis and shorter survival. Multivariate survival analysis identified high EpCAM expression as an independent prognostic factor. Ectopic EpCAM expression in NPC cells promoted epithelial-mesenchymal transition (EMT), induced a cancer stem cell (CSC)-like phenotype, and enhanced metastasis in vitro and in vivo without an effect on cell proliferation. Notably, EpCAM overexpression reduced PTEN expression and increased the level of AKT, mTOR, p70S6K and 4EBP1 phosphorylation. Correspondingly, an AKT inhibitor and rapamycin blocked the effect of EpCAM on NPC cell invasion and stem-like phenotypes, and siRNA targeting PTEN rescued the oncogenic activities in EpCAM knockdown NPC cells. Our data demonstrate that EpCAM regulates EMT, stemness and metastasis of NPC cells via the PTEN/AKT/mTOR pathway.

Nasopharyngeal carcinoma (NPC) is particularly common in Southern China and Southeastern Asia, where the incidence peaks at 50 cases per 100,000 people per year^1,2^. NPC exhibits the highest invasive and metastasis potential among head and neck cancers, with 15–30% of patients developing distant metastasis despite high sensitivity of the tumour to radiotherapy^3^. The prognosis for advanced NPC is poor, with a 5-year survival rate ranging from 50 to 70%, and distant metastasis is the main obstacle in the current clinical management of NPC^4,5^. Therefore, better treatment strategies will ultimately require a clearer understanding of the molecular basis of NPC metastasis.

EpCAM (epithelial cell adhesion molecule; CD326 (cluster of differentiation 326)) was originally identified as a novel tumour-specific cell surface antigen after immunisation of mice with cancer cells in 1970s, and was later defined as a cell–cell adhesion molecule^6,7^. EpCAM is a type I transmembrane glycoprotein with an ectodomain, one transmembrane domain, and a cytoplasmic domain of 26 residues^8,9^. This glycoprotein is specifically expressed in epithelial tissue and overexpressed in a large variety of human epithelial-derived neoplasms, including cancer of the tongue^10^, thyroid^11^, prostate^12–14^, oesophagus^15^, liver^16,17^, colon^18^, breast^19,20^, ovary^21^, pancreas^22^, gallbladder^23^, lung^24^, stomach^25^ and kidney^26^. Recent studies have revealed that EpCAM is involved in cell signalling, migration, proliferation and differentiation, as well as in metastasis and cancer stem cells^27^. However, conflicting data have been published describing EpCAM in some carcinoma types as a tumour suppressive protein that is associated with improved patient survival^11,28–31^. Whether EpCAM acts as a tumour suppressive gene or as an oncogene might depend on the cell type and microenvironment.

Although EpCAM is one of the best studied cancer-associated antigens, its expression profile, biological function and clinical significance in NPC have not been reported until now. In our previous study, deep sequencing of the human NPC cell lines CNE2 and C666-1 and the immortalised nasopharyngeal epithelial cell line NP69 was performed using Illumina Hiseq 2500 with the aim of characterising aberrant transcript expression that contributes to NPC oncogenesis (unpublished). Among the most highly upregulated genes, the EpCAM gene showed dramatically elevated expression in C666-1 and CNE2 cells compared with the NP69 cells (logFC = 6.25 and 6.00, respectively) (Supplementary Table 1). Therefore, the aim of this study was to explore the expression profile of EpCAM and its role in NPC aggressiveness.

## Results

### EpCAM is frequently upregulated in NPC tissues and cells

**Fig. 1:**
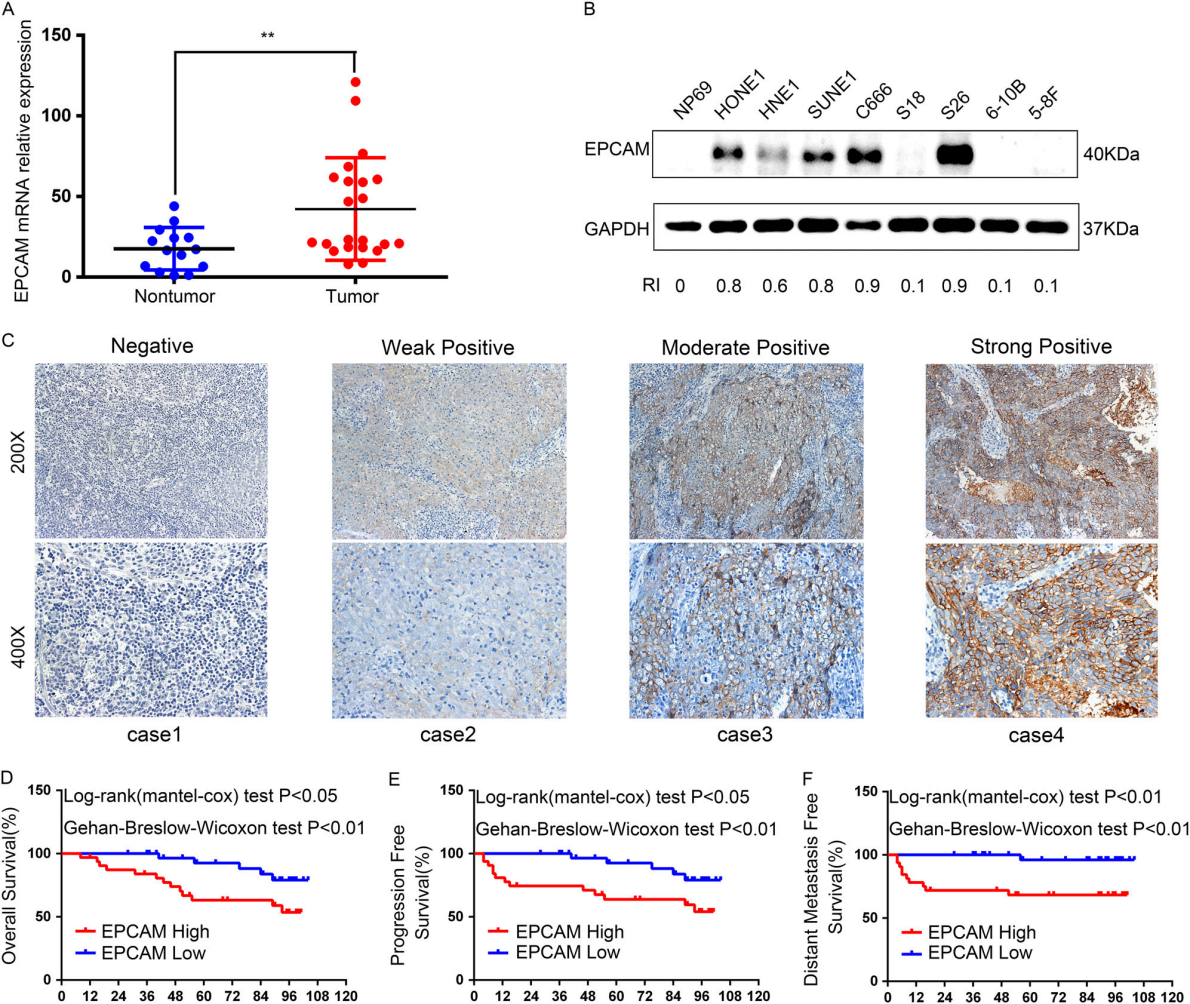
EpCAM overexpression is frequently detected in NPC tissues and predicts a poor prognosis. **a** The EpCAM mRNA level was elevated in NPC tumour tissues (*n* = 22) compared with non-cancerous nasopharyngitis tissues (*n* = 14) based on qRT-PCR analysis (*P* < 0.01, independent Student’s *t*-test). **b** Protein expression levels of EpCAM in a panel of NPC cell lines analysed by western blot. GAPDH is used as a loading control. **c** Differential EpCAM protein expression in representative NPC tumours, determined by immunohistochemical staining. **d**–**f** Kaplan–Meier survival curve analysis indicated that NPC patients with higher EpCAM expression had shortened overall survival (OS), progression-free survival (PFS) and distant-metastasis-free survival (DMFS) compared with patients with low EpCAM expression (log-rank test)

In the current study, EpCAM expression levels were evaluated in 22 snap-frozen NPC tissues and 14 non-cancerous nasopharyngitis (NP) tissues using quantitative real-time PCR (qRT-PCR), and the results showed that EpCAM was significantly upregulated in tumour tissues in comparison with non-tumour tissues (*P* < 0.01) (Fig. 1a). Moreover, western blotting analysis revealed an obviously higher level of EpCAM expression in HONE1, SUNE1, C666-1 and S-26 cell lines, whereas the normal epithelial NP69 cell line and the other four NPC cell lines (HNE1, S-18, 6–10B and 5–8F) showed undetectable or very low levels of endogenous EpCAM expression (Fig. 1b). In the following experiments, S-18 and 6–10B cells were used to generate EpCAM-overexpressing cell lines, and HONE1 cells were used for targeted EpCAM knockdown.

### EpCAM overexpression is correlated with distant metastasis and poor prognosis in NPC patients

To explore the clinical significance of EpCAM expression in NPC patients, we performed immunohistochemistry (IHC) to measure the expression of EpCAM in 64 formalin-fixed paraffin-embedded NPC samples. Representative images of NPC tissues with different IHC staining intensities are shown in Fig. 1c. Absence of EpCAM was observed in 2 NPC cases (3.1%). The total IHC staining scores for EpCAM in these NPC samples ranged from 0 to 3. The median of the IHC scores (0.9) was used as the threshold of EpCAM expression in this study, and EpCAM overexpression was defined as a total score of >0.9. Thus, 28 out of the 64 (43.75%) NPC samples were identified as high EpCAM expressing (staining index >0.9). The clinicopathological characteristics of the 64 NPC patients are summarised in Table 1. The metastasis rate was significantly higher in EpCAM high-expression patients (42.9%, 12/28) than in EpCAM low-expression patients (8.3%, 3/36) (*χ*^2^ = 10.461, *P* = 0.001). No significant associations were found between EpCAM expression and other clinicopathological features.

**Table 1:** Association of EpCAM expression and patient clinicopathological characteristics in NPC tissues

Characteristics	No.	EpCAM expression level		*P-*value (*χ*^2^-test)
		Low	High	
Gender				
Male	47	24 (51.1%)	23 (48.9%)	0.164
Female	17	12 (70.6%)	5 (29.4%)	
Age				
<44	32	17 (53.1%)	15 (46.9%)	0.200
≥44	32	22 (68.8%)	10 (31.2%)	
DNA copy				
≤4000	40	22 (55.0%)	18 (45.0%)	0.795
>4000	24	14 (58.3%)	10 (41.7%)	
EA/IgA				
<1:10	14	8 (57.1%)	6 (42.9%)	0.939
≥1:10	50	28 (56.0%)	22 (44.0%)	
VCA/IgA				
<1:80	10	4 (40.0%)	6 (60.0%)	0.259
≥1:80	54	32 (59.3%)	22 (40.7%)	
T-stage				
T1–2	20	11 (55.0%)	9 (45.0%)	0.892
T3–4	44	25 (56.8%)	19 (43.2%)	
N-stage				
N0–1	31	19 (61.3%)	12 (38.7%)	0.431
N2–3	33	17 (51.5%)	16 (48.5%)	
Clinical staging				
I–II	4	3 (75.0%)	1 (25.0%)	0.435
III–IV	60	33 (55.0%)	27 (45.0%)	
Distant metastasis				
Yes	15	3 (28.6%)	12 (80.0%)	0.001*
No	49	33 (67.3%)	16 (32.7%)	

Statistically significant difference (*P*<0.05) was indicated in bold letters.

For survival analysis, 64 patients who were monitored for at least 3 years after treatment or until death were included, with a median observation time of 89 months (range 8–104 months). Kaplan–Meier survival curves and log-rank test survival analysis showed that the overall survival (OS), progression-free survival (PFS) and distant-metastasis-free survival (DMFS) of NPC patients with high levels of EpCAM was significantly poorer than that of patients with low levels of EpCAM (*P* < 0.05; Fig. 1d–f). Multivariate Cox regression analysis demonstrated that EpCAM expression was an independent predictor of OS (HR, 1.01; 95% CI, 1.003–1.016; *P* = 0.003), PFS (HR, 1.01; 95% CI, 1.003–1.016; *P* = 0.003) and DMFS (HR, 1.011; 95% CI, 1.002–1.019; *P* = 0.014) (Table 2).

**Table 2:** Univariate and multivariate analyses of overall survival (OS), progression-free survival (PFS) and distant-metastasis-free survival (DMFS) in patients with NPC

	Variables	Univariate analysis			Multivariate analysis		
		HR	95% CI	*P*-value	HR	95% CI	*P*-value
OS	Gender (male vs. female)	1.207	0.389–3.746	0.745			
	Age (≥44 vs.<44)	2.418	0.839–6.971	0.102			
	EBV-DNA copy (4000>vs. ≤4000)	1.124	0.408–3.095	0.821			
	EA/IgA (≥1:10 vs. <1:10)	1.206	0.343–4.234	0.770			
	VCA/IgA (≥1:80 vs. <1:80)	1.104	0.25–4.876	0.896			
	T-stage (T3–4 vs. T1–2)	2.256	0.642–7.926	0.205			
	N-stage (N2–3 vs. N0–1)	0.647	0.235–1.784	0.400			
	Clinical staging (III+IV vs. I+II)	2.74	1.064–7.079	0.037	3.033	1.278–7.197	0.012
	EpCAM (high vs. low level)	1.008	1.001–1.014	0.018	1.01	1.003–1.016	0.003
PFS	Gender (male vs. female)	1.385	0.455–4.211	0.566			
	Age (≥44 vs. <44)	1.728	0.715–4.175	0.224			
	EBV-DNA copy (4000>vs. ≤4000)	0.934	0.350–2.489	0.891			
	EA/IgA (≥1:10 vs. <1:10)	0.986	0.324–2.997	0.980			
	VCA/IgA (≥1:80 vs. <1:80)	0.808	0.233–2.802	0.737			
	T-stage (T3–4 vs. T1–2)	2.621	0.758–9.061	0.128			
	N-stage (N2–3 vs. N0–1)	2.621	0.758–9.061	0.128			
	Clinical staging (III+IV vs. I+II)	3.336	1.343–8.287	0.009	3.117	1.314–7.394	0.010
	EpCAM (high vs. low level)	1.008	1.003–1.014	0.005	1.01	1.003–1.016	0.003
DMFS	Gender (male vs. memale)	3.705	0.474–28.947	0.212			
	Age (≥44 vs. <44)	1.600	0.653–3.921	0.304			
	EBV-DNA copy (4000>vs. ≤4000)	1.487	0.453–4.875	0.513			
	EA/IgA (≥1:10 vs. <1:10)	0.735	0.195–2.771	0.650			
	VCA/IgA (≥1:80 vs. <1:80)	0.767	0.166–3.550	0.734			
	T-stage (T3–4 vs. T1–2)	4.818	0.616–37.675	0.134			
	N-stage (N2–3 vs. N0–1)	1.765	0.516–6.034	0.365			
	Clinical staging (III+IV vs. I+II)	18.823	2.423–146.215	0.005	14.252	1.828–111.123	0.011
	EpCAM (high vs. low level)	1.012	1.004–1.021	0.003	1.011	1.002–1.019	0.014

Collectively, these data showed that EpCAM was associated with disease progression and prognosis of NPC.

### EpCAM expression in NPC cells has no effect on cell growth and proliferation

**Fig. 2:**
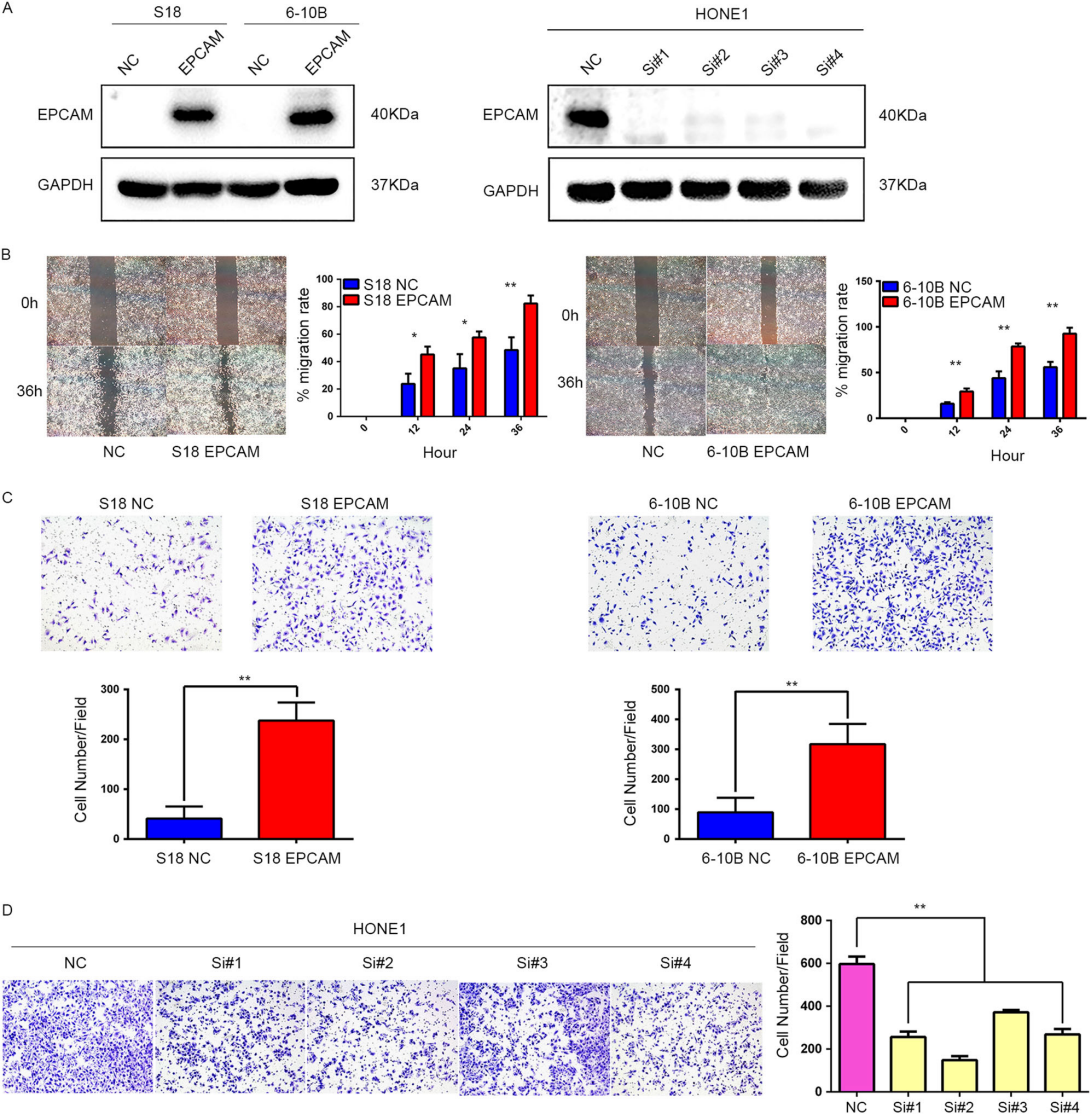
EpCAM promotes NPC cell migration and invasion. **a** EpCAM overexpression efficiency in S18-EpCAM and 6–10B-EpCAM cells (left) and knockdown of endogenous EpCAM expression in HONE1 cells via siRNA transfection (right) was confirmed by western blot. **b** Wound healing assays showed that S18-EpCAM and 6–10B-EpCAM cells had higher motility compared with control cells. Left, representative images taken 0 h and 36 h after the scratch was made. Right, quantification of cell migration results, which are expressed as the mean ± S.D. of three independent experiments. *P < 0.05 and **P < 0.01 by Student’s *t-*test. **c** Cell invasion was evaluated using a Matrigel invasion chamber. EpCAM overexpression increased the invasive capacity of S-18 and 6–10B cells. Upper, representative images of cells that migrated through the PET membrane (magnification ×200). Lower, quantification of cell invasion data. The results are expressed as the mean ± S.D. of three independent experiments. **P < 0.01 by Student’s *t-*test. **d** Inhibition of EpCAM expression leads to a marked reduction in HONE1 cell invasiveness

NPC cell lines stably expressing EpCAM were generated from S18 and 6–10B cells using recombinant lentivirus encoding EpCAM or a negative control lentivirus (NC). To further explore the role of endogenous EpCAM in NPC cells, EpCAM was silenced in HONE1 cells by each of four specific siRNA oligos against EpCAM. Scrambled siRNA was used as a control. Upregulation and knockdown of EpCAM expression was confirmed by western blot analysis (Fig. 2a).

CCK8 assays, colony formation assays and cell cycle analysis showed no significant differences between EpCAM-overexpressing cells and the control cells (Supplementary Fig. S1 A–C). To further explore the role of EpCAM in NPC cell growth in vivo, growth of tumours derived from S-18-EpCAM cells was examined in nude mice. Subcutaneous xenograft formation was observed in all mice in the S-18-EpCAM group (*n* = 4) and in the control group (*n* = 4), while no significant difference in tumour number or size was found between EpCAM-overexpressing and control groups (Supplementary Fig. S1 D). EpCAM overexpression was confirmed by IHC in xenograft tumours derived from S18-EpCAM cells. These results confirm that EpCAM has no effect on NPC cell proliferation in vitro and in vivo.

Our results were inconsistent with previous studies reporting that EpCAM could stimulate cell cycle progression and proliferation by increasing the expression of c-myc and cyclin D1^31,32^. To additionally test the potential impact of EpCAM on NPC cell proliferation, we detected the effect of EpCAM on the expression of c-myc and cyclin D1 using a western blot assay. The results showed that neither EpCAM overexpression or knockdown could affect the expression levels of c-myc and cyclin D1 (Supplementary Fig. S1 E).

### Upregulation of EpCAM promotes NPC cell epithelial-mesenchymal transition and in vivo metastasis

**Fig. 3:**
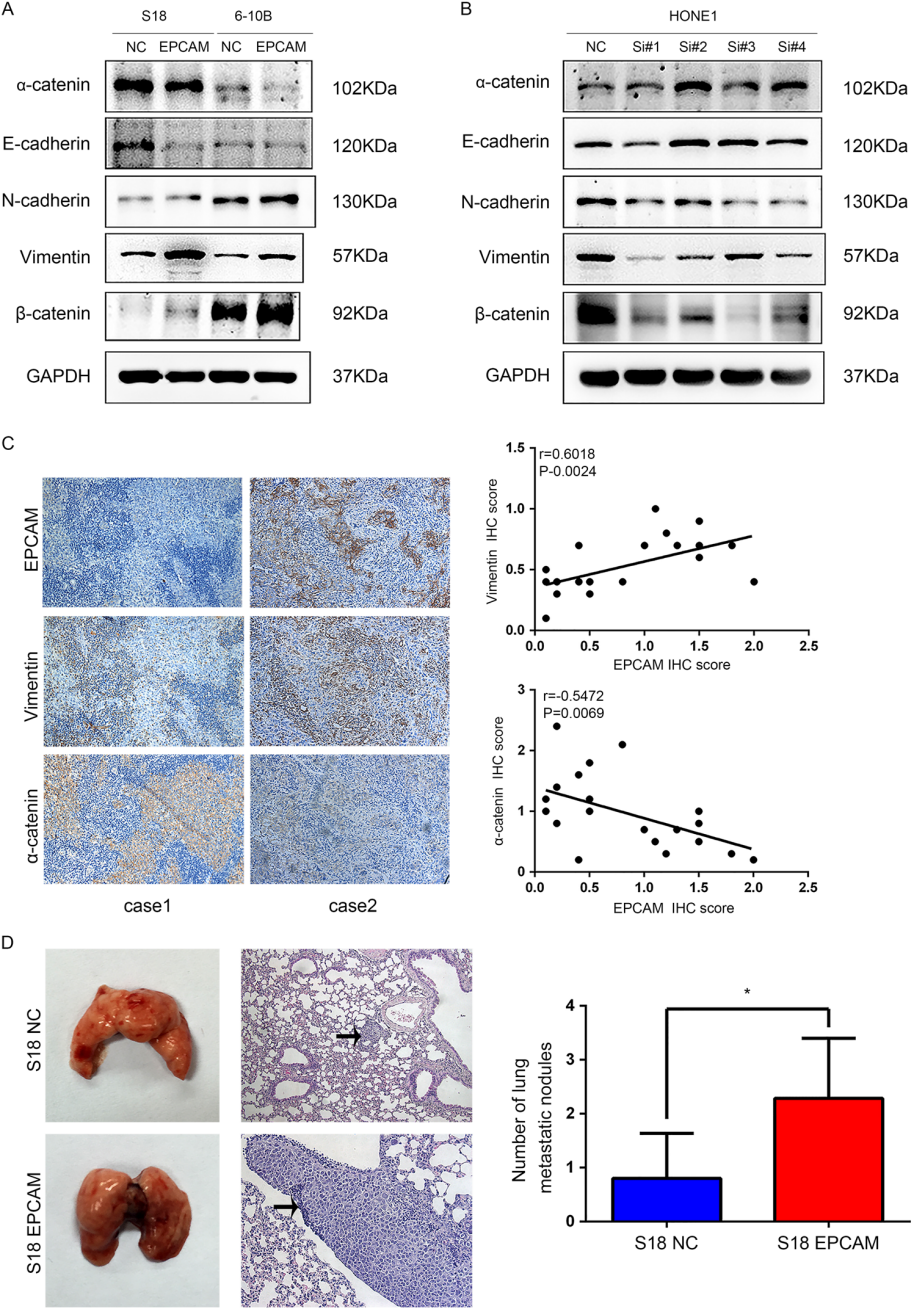
EpCAM expression promotes NPC cell epithelial-mesenchymal transition and metastasis in nude mice. **a** Western blotting reveals reduced expression of epithelial makers (E-cadherin and α-catenin) and an increased expression of mesenchymal markers (N-cadherin, Vimentin and β-catenin) in EpCAM-expressing S-18 and 6–10B cells in comparison with the vector controls. **b** Western blotting assay showed increased levels of E-cadherin and α-catenin and decreased levels of N-cadherin, Vimentin and β-catenin in EpCAM knockdown HONE1 cells compared with that in shNC-treated cells. **c** Correlation between EpCAM and α-catenin and Vimentin expression in NPC tissues (*n* = 23). Left, immunohistochemical staining of representative NPC samples (magnification ×200). Right, Pearson correlation coefficient was calculated on the basis of the relative protein expression levels (IHC scores) of EpCAM, α-catenin and Vimentin in NPC samples. **d** Overexpression of EpCAM promoted S-18 cell invasion and metastasis in athymic nude mice in vivo. Left, two lung samples showing metastatic nodules; middle, examples of haematoxylin and eosin staining in two lung samples originating from S-18-vector and S-18-EpCAM cell-injected mice (magnification ×400); right, number of metastatic nodules formed in the lungs of mice 8 weeks after tail vein injection of S-18-vector and S-18-EpCAM cells (7 mice in each group; *P* < 0.05, independent Student’s *t-*test)

In light of the obvious connection between EpCAM expression and distant metastasis in clinical samples, we examined the potential regulation of EpCAM on EMT, which is considered to be a critical process in cancer metastasis. To determine whether EpCAM overexpression induces EMT phenotypes in NPC cells, we first investigated the effect of EpCAM on invasion and metastasis. In a wound healing assay, the gap filling was remarkably accelerated in the S18-EpCAM and 6–10B-EpCAM cells compared with the control cells (Fig. 2b). In a Matrigel invasion transwell assay, an increased number of invading cells, from 41 to 237 (*P* < 0.01), was observed in the S18-EpCAM cell line, and the number of invading cells in the 6–10B cell line increased from 89 to 317 (*P* < 0.01) (Fig. 2c). On the other hand, knockdown of EpCAM in HONE1 cells with four different siRNA oligos decreased the number of invading cells from 596 to 256, 148, 371 and 268, depending on the siRNA oligo employed (Fig. 2d). Western blotting analysis demonstrated that the mesenchymal markers N-cadherin, Vimentin and β-catenin were significantly upregulated and the expression of the epithelial markers α-catenin and E-cadherin was decreased in the EpCAM-expressing S18 and 6–10B cells (Fig. 3a). The opposite results were obtained in EpCAM-silenced HONE1 cells (Fig. 3b). To further verify the correlation between EpCAM expression levels and EMT markers in clinical NPC samples, we stained consecutive sections of NPC tissues from 23 cases using IHC and correlated the EpCAM staining data with that of EMT markers. Significant linear correlations were found between EpCAM and Vimentin (*R* = 0.6018, *P* = 0.0024) and between EpCAM and α-catenin (*R* = 0.5472, *P* = 0.0069) in NPC tissues (Fig. 3c).

To further confirm metastatic potential in vivo, S18-EpCAM and NC cells were injected into nude mice through the tail vein. Eight weeks after injection, histological analyses confirmed a higher incidence of lung metastasis in mice injected with S18-EpCAM cells (*n* = 7) compared with mice in the control group (*n* = 7) (*P* < 0.05, Fig. 3d).

Collectively, our results confirmed that upregulation of EpCAM promotes NPC cell metastasis in vitro and in vivo.

### EpCAM overexpression mediates stem-like properties of NPC cells

**Fig. 4:**
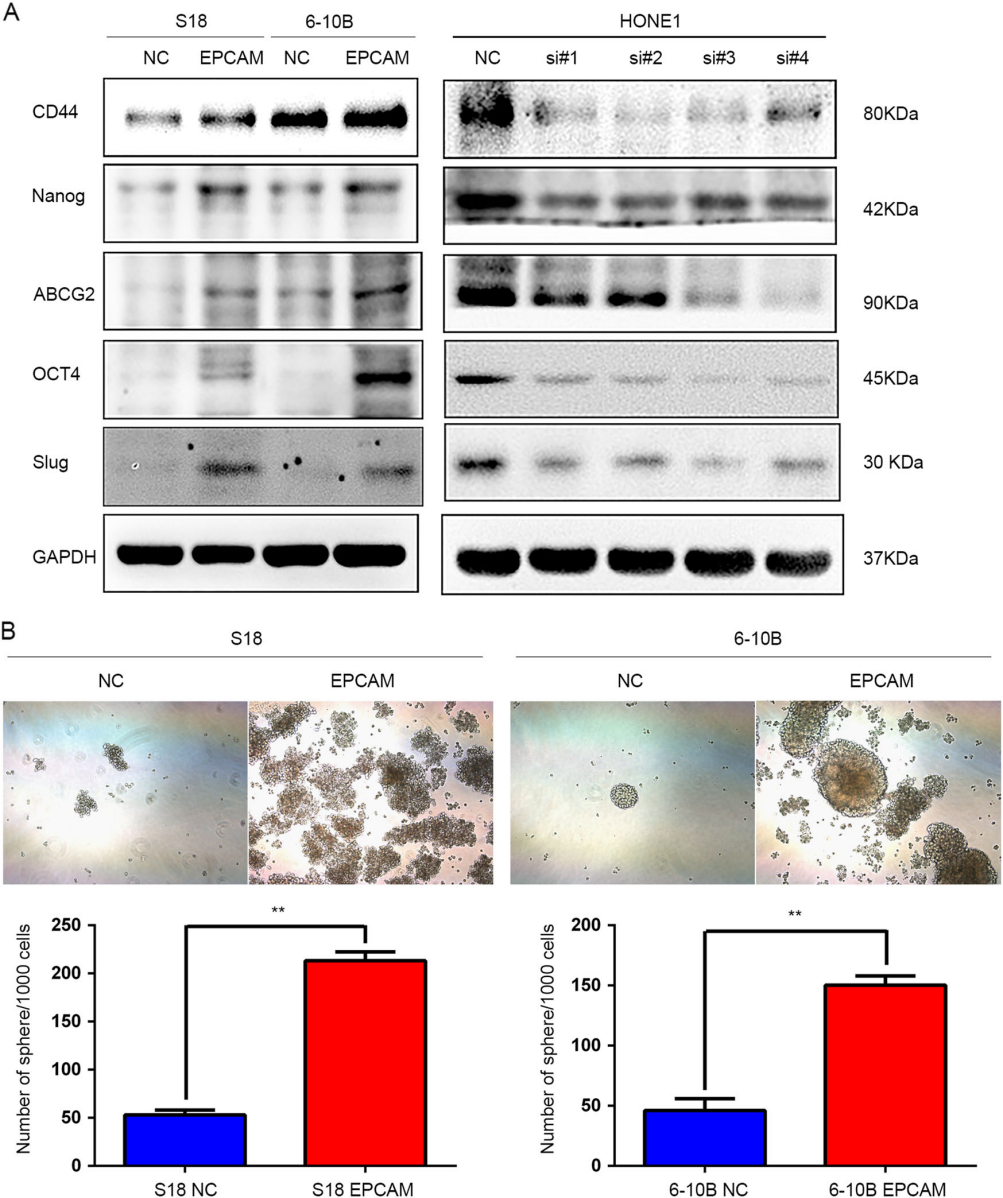
EpCAM overexpression mediates stem-like properties of NPC cells. **a** Western blot analysis showed that the expression levels of the CSC biomarkers CD44, OCT4, Nanog and ABCG2, as well as the EMT regulatory factor Slug were significantly enhanced in the EpCAM-expressing S-18 and 6–10B cells but suppressed in EpCAM-depleted HONE1 cells compared with control cells. **b** Representative light microscopy images of stem-like spheres after ectopic EpCAM expression in S-18 and 6–10B cells. The number of stem-like spheres formed in each group was determined in triplicate plates. All values are expressed as the mean ± SEM. Overexpression of EpCAM significantly enhanced sphere formation of NPC cells (**P < 0.01)

To determine if EpCAM expression regulates the stem-like phenotype, the expression of stem cell biomarkers was examined using western blot analysis. We demonstrated that the expression levels of the cancer stem-like cell markers CD44, OCT4, Nanog and ABCG2, as well as the EMT regulatory factor Slug were significantly enhanced in EpCAM-overexpressing cells and reduced in EpCAM-depleted HONE1 cells compared with control cells (Fig. 4a). Moreover, a sphere formation assay showed that a significantly greater number of spheres formed in culture when EpCAM was overexpressed (*P* < 0.01, Fig. 4b), and the spheres had a greater range of sizes. Collectively, these results suggest that EpCAM has a role in the transition to a stem-like phenotype in NPC cells.

### EpCAM induces EMT and stem-like properties by regulating the PTEN/AKT/mTOR signalling pathway in NPC cells

**Fig. 5:**
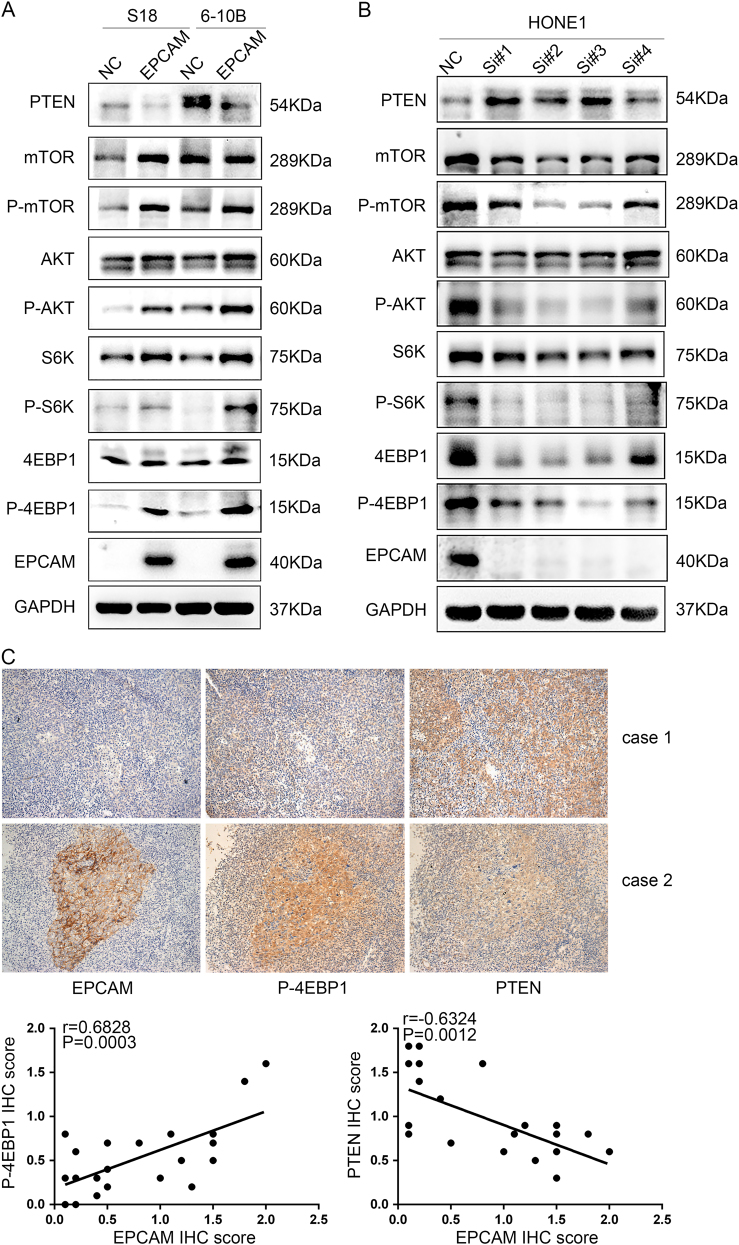
EpCAM activates the PTEN/AKT/mTOR signalling pathway in NPC cells. **a**, **b** Western blotting analysis revealed that EpCAM overexpression decreases PTEN expression and increases phosphorylated protein levels of mTOR, AKT, S6K and 4EBP1 (p-mTOR, p-AKT, p-p70S6k and p-4EBP1) in S-18 and 6–10B cells **a**, whereas knockdown of EpCAM increased PTEN and decreased the protein levels of p-mTOR, p-AKT, p-p70S6k and p-4EBP1 in HONE1 cells **b**. GAPDH was used as a loading control. **c** Correlation between EpCAM and PTEN and p4E-BP1 expression in NPC tissues (*n* = 23). Immunohistochemical staining of representative NPC samples is shown (magnification ×200). Pearson correlation coefficient was calculated on the basis of the relative protein expression levels (IHC scores) of EpCAM, PTEN and p4E-BP1 in NPC samples

Because the PI3K/AKT pathway regulates metastasis and is frequently activated in NPC, we examined the effect of EpCAM expression on AKT signalling activity. A substantial increase in the levels of key molecules in the AKT signalling pathway, specifically, phosphorylated (active) AKT, mTOR, P70S6K and 4EBP but not the total amount of these proteins, was observed in S-18 and 6–10B cells overexpressing EpCAM (Fig. 5a). Conversely, siRNA-mediated depletion of EpCAM in HONE1 cells decreased the levels of phosphorylated AKT, mTOR, P70S6K and 4EBP1 (Fig. 5b). Moreover, the expression level of PTEN, a negative regulator of AKT signalling, was decreased in EpCAM-overexpressing cells and increased in EpCAM-depleted HONE1 cells (Fig. 5a, b). IHC analysis of 23 NPC cases identified a positive correlation between the EpCAM expression level and phosphorylated 4EBP1 (*R* = 0.6828, *P* = 0.0003) and a negative correlation between EpCAM and PTEN (*R* = 0.6324, *P* = 0.0012) (Fig. 5c).

**Fig. 6:**
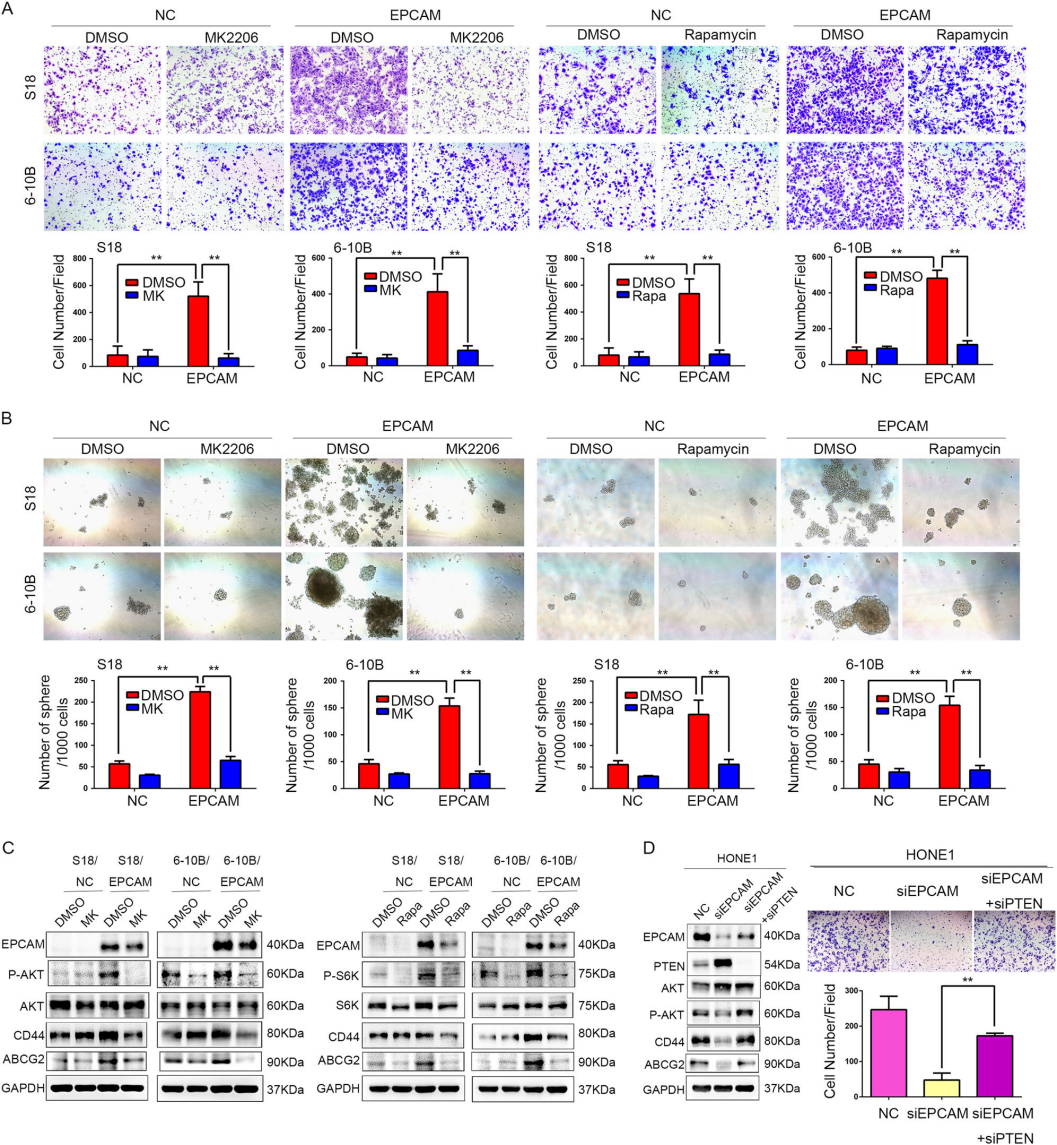
Inhibitors of the PTEN/AKT/mTOR pathway abolished the effect of EpCAM on NPC cell invasion and stem-like properties. **a** The invasive abilities of S-18 and 6–10B cells expressing EpCAM or the empty vector were assessed with transwell assays after pretreatment with or without 5 μM MK2206 (AKT inhibitor) (left) or 100 nM rapamycin (right). **b** The sphere formation efficiency of S-18 and 6–10B cells expressing EpCAM or the empty vector was evaluated after pretreatment with or without 5 μM MK2206 (left) or 100 nM rapamycin (right). **c** EpCAM-induced CD44 and ABCG2 expression was diminished by MK2206 and rapamycin treatment. **d** Suppression of PTEN expression by siRNA restored the reduced invasion and CD44 and ABCG2 expression in EpCAM-depleted HONE1 cells. Transwell assays were performed with HONE1 cells transfected with siRNA-EpCAM, siRNA-EpCAM plus siRNA-PTEN, or NC. Suppression of PTEN expression by siRNA in EpCAM-repressed cells rescued AKT/mTOR activities

To further determine whether activation of the PTEN/AKT/mTOR signalling pathway is important for the oncogenic activity of EpCAM in NPC cells, EpCAM-expressing S-18 and 6–10B cells were treated with the pharmacological AKT inhibitor MK2206 or the mTOR inhibitor rapamycin. Treatment with the AKT inhibitor MK2206 or rapamycin almost completely abrogated the enhanced invasion and sphere formation ability induced by EpCAM overexpression (Fig. 6a, b). EpCAM-stimulated CD44 and ABCG2 expression were also blunted by MK2206 and rapamycin treatment (Fig. 6c). In addition, suppression of PTEN by siRNA in EpCAM-depleted cells rescued AKT signalling activities in NPC cells (Fig. 6d). Moreover, the reduced invasion ability in HONE1 cells resulting from EpCAM knockdown was restored by suppression of PTEN expression (Fig. 6d). All of these findings confirmed that the EpCAM-induced invasiveness and stemness of NPC cells depends on PTEN/AKT/mTOR signalling. We also found that treatment with the AKT inhibitor or with rapamycin reduced the EpCAM expression level, while suppression of PTEN increased EpCAM expression to some extent, which suggest that AKT/mTOR signalling activation might upregulate EpCAM expression.

To confirm whether activation of the PTEN/AKT/mTOR signalling pathway is required for the EMT induction activity of EpCAM in NPC cells, EpCAM-expressing 6–10B cells were treated with MK2206 or rapamycin. The induced expression of Vimentin and Slug by EpCAM overexpression was abrogated by treatment of MK2206 or rapamycin (Supplementary Fig. S2A, B). On the other hand, the decreased expression of Vimentin and Slug in HONE1 cells resulting from EpCAM knockdown was recovered by suppression of PTEN expression (Supplementary Fig. S2C).

## Discussion

Our previous study revealed that EpCAM was upregulated in NPC cell lines. In the present study, EpCAM mRNA and protein overexpression was confirmed in NPC biopsies. Moreover, increased levels of EpCAM correlated with a high metastatic potential and shorter survival of NPC patients. Cox multivariate regression analysis showed that EpCAM is an independent prognostic predictor of poor survival in NPC patients. EpCAM overexpression was demonstrated to enhance S-18 and 6–10B cell invasion and migration ability in transwell and wound healing assays and promoted in vivo metastasis. In contrast, knockdown of EpCAM expression in HONE1 cells weakened their invasive capability. Our data are the first to show that EpCAM is overexpressed in NPC and promotes metastasis in NPC patients.

Previous studies have shown that EpCAM can directly stimulate cell cycle progression and proliferation through upregulation of c-myc and subsequent upregulation of the cell cycle-related proteins cyclin A and E^32^. EpCAM has also been shown to regulate the expression of cyclin D1 at the transcriptional level^33^. However, in the present study, EpCAM was shown to have no effect on cell cycle and cellular growth of NPC cells in vitro or in vivo according to the cell viability assay, colony formation assay, cell cycle analysis by flow cytometry and subcutaneous xenograft model. Furthermore, no change was found in the expression level of c-myc and cyclin D1 when EpCAM was overexpressed or reduced in NPC cell lines. The discrepancy between our findings and previous reports may be due to the different cell type used in the respective studies, and further experiments need to be performed to elucidate the underlying mechanism.

EMT is an essential early step that plays an important role in the process of tumour invasiveness and metastasis. During cancer progression, EMT can endow cancer cells with self-renewal capabilities and lead them to express stem cell-associated cell-surface markers, which contribute to therapy resistance, tumour relapse and metastasis. Many studies have demonstrated that EpCAM might foster higher cell plasticity and motility by weakening cadherin-mediated cell–cell adhesion in epithelial malignancies^34^. EpCAM has been widely used as a marker of CSCs because of its ubiquitous overexpression in CSCs that originate from epithelial lesions^35^. However, the molecular changes following EpCAM expression have not been clearly understood until now. Here, we showed that EpCAM overexpression increases the mRNA and protein levels of the EMT transcription factors Slug, promotes EMT, and enhances the expression of stem cell markers and sphere formation in NPC cells.

Since the mechanistic insights into the functions of EpCAM have only very recently emerged, the function of EpCAM and its regulatory mechanism are still largely unknown. In the present study, we demonstrate that EpCAM promotes metastasis, EMT and stem-like properties via PTEN/AKT/mTOR signalling. Aberrant activation of AKT/mTOR signalling frequently occurs in NPC and is correlated with metastasis and poor prognosis^36,37^. The widely known negative regulator of PI3K/AKT signalling, PTEN, was significantly downregulated in NPC^38,39^. The AKT/mTOR pathway is known to promote invasion and metastasis through regulation of EMT and stem cell features^40,41^. The EMT regulatory factor Slug has been reported to be a downstream target of PI3K/AKT, and PI3K/AKT signalling is essential for enabling EMT in response to upregulation of Slug^42^. Here, we show that EpCAM overexpression inhibits PTEN expression and activates AKT/mTOR signalling in NPC cells. The mechanistic investigation using inhibitors targeting AKT and mTOR revealed that AKT/mTOR signalling activation is important for the oncogenic effect of EpCAM on cell invasion and stem-like properties of cancer cells.

A previous study showed that intramembrane proteolysis of EpCAM results in nuclear transport of the intracellular domain (ICD) of EpCAM (EpICD), which interacts with β-catenin and affects Wnt-target gene transcription^43^. A study of hepatocellular carcinoma showed that activation of the Wnt/β-catenin pathway upregulates EpCAM expression. Thus, EpICD may create a positive-feedback loop that modulates EpCAM expression^44^. Interestingly, our results showed that activation of the AKT/mTOR pathway could also upregulate EpCAM expression (Fig. 6c), indicating a positive-feedback loop involving EpCAM-AKT/mTOR-EpCAM might exist. The double-positive feedback loop mediated by the Wnt and AKT/mTOR pathways, two critical signalling pathways in NPC cells, might be used to keep EpCAM at a high level and promote tumour progression, although further experiments are needed to verify this hypothesis.

In conclusion, we have demonstrated that EpCAM is upregulated in NPC and plays a pro-metastatic role. Moreover, we revealed that EpCAM promotes EMT, stemness and metastasis of NPC cells via the PTEN/AKT/mTOR signalling pathway. We propose that EpCAM may serve as a potential prognostic marker of NPC.

## Materials and methods

### Patients and samples

In total, 64 formalin-fixed, paraffin-embedded primary NPC specimens obtained from the Department of Pathology in the Sun Yat-Sen University Cancer Center (SYSUCC) were used for the immunohistochemistry assay. All the patients were first diagnosed with NPC both histologically and clinically between 2007 and 2008 according to the TNM staging of the International Union against Cancer. There were 17 males and 47 females with a median age of 44 years, ranging from 17 to 70. Clinical information associated with the samples is summarised in Table 1. Additionally, 22 cases of fresh clinical NPC tissues and 14 non-cancerous nasopharyngitis (NP) tissues were collected from SYSUCC and stored at −80 °C before qRT-PCR analysis. None of the patients were subjected to radiotherapy or chemotherapy before biopsy sampling. The Research Ethics Committee of SYSUCC approved our study, and all patients provided written informed consent.

### Cell lines

The human NPC cell lines HONE1, HNE1, SUNE1, C666-1, S18, S26, 6–10B and 5–8 F were maintained in RPMI-1640 medium (Gibco, Shanghai, China) supplemented with 10% foetal bovine serum (Gibco, US). The normal nasopharyngeal epithelium cell line NP69 was maintained in defined-KSFM medium supplemented with EGF (Invitrogen, Carlsbad, CA). All cell lines were cultured in a humidified atmosphere of 5% CO_2_ at 37 °C.

### Antibodies and reagents

Antibodies against GAPDH, EpCAM (D9S3P), AKT, P-AKT(S473), mTOR, P-mTOR, S6K, P-S6K(T398), 4EBP1, P-4EBP1, PTEN, CD44, Oct4, Nanog, c-myc, cyclin D1 and Slug were purchased from Cell Signaling Technology. ABCG2 antibody was obtained from Santa Cruz Biotechnology. Mouse monoclonal antibodies against human α-catenin, E-cadherin, fibronectin and Vimentin were purchased from BD Biosciences. GAPDH antibody was used for a normalised control. Rapamycin and the AKT inhibitor MK2206 were purchased from Selleck Chemicals. To test the effect of AKT/mTOR activation on cell invasiveness, sphere formation ability and gene expression induced by EpCAM expression, NPC cells were pretreated with the AKT inhibitor MK2206 (5 μM) or Rapamycin (100 nM) for 24 h before performing the following experiments and assays.

### Immunohistochemistry staining

The immunohistochemistry (IHC) study was performed using a standard streptavidin-biotin-peroxidase complex method described previously. Then, the staining results were evaluated and scored independently by two pathologists. The intensities were graded as 0 (negative), 1 (weakly positive), 2 (moderately positive) and 3 (strongly positive), and the estimated fraction of positively stained tumour cells was evaluated by percentage from 0 to 100%. Finally, the intensity and proportion score were multiplied to determine the total IHC score of each section.

### Quantitative real-time PCR (qRT-PCR)

Total RNA was extracted with TRIzol (Invitrogen, USA) according to the manufacturer’s instructions. For the gene expression test, 1 μg of total RNA was used to synthesis cDNA using a PrimeScript RT reagent kit (Promega, Madison, WI, USA). Then, qRT-PCR was carried out in a total volume of 15 µl using Platinum SYBR Green qPCR SuperMix-UDG (Invitrogen, USA) and a Bio-Rad CFX96 Real-Time PCR Detection System following the manufacturer’s protocol. The specific primer sequences used in this study are listed below: EpCAM sense 5′-AATCGTCAATGCCAGTGTACTT-3′, EpCAM anti-sense 5′- TCTCATCGCAGTCAGGATCATAA-3′; GAPDH sense 5′- CTCCTCCTGTTCGACAGTCAGC-3′ and GAPDH anti-sense 5′- CCCAATACGACCAAATCCGTT-3′. Each sample was repeated across three independent tests.

### Generation of NPC cell lines with stable expression of EpCAM

EpCAM expression lentiviral vector and empty vector were purchased from GenePharma (Shanghai, China). S18 and 6–10B cells were infected with recombinant lentivirus-transducing units plus 8 mg/mL polybrene (Sigma, St Louis, Missouri, USA) according to the manufacturer’s instructions. Cells were selected using 4 μg/mL puromycin for 2 weeks, and stable EpCAM expression and control cell lines were obtained.

### RNA interference

Small interfering RNA (siRNA) duplexes targeting EpCAM (siRNA459, siRNA738, siRNA980 and siRNA1082) and the negative control (NC) siRNA were designed and synthesised by GenePharma (Shanghai, China). siRNA mix against PTEN (5′-TGCAGCAATTCACTGTAAA-3′ and 5′-GAGCGTGCAGATAATGACA-3′) and control RNA were purchased from Guangzhou RiboBio (www.ribobio.com). Cell transfection was achieved using Lipofectamine 3000 (Invitrogen, USA) according to the manufacturer’s instructions. Briefly, 100 nM siRNA duplex was incubated with cell lines for 72 h for each transfection, after which the following experiments and assays were performed.

### Western blotting

Cells or tissue homogenates were lysed using RIPA buffer containing PMSF (RIPA:PMSF = 100:1). Then, after 30 min on ice and centrifugation at 4 °C, 14,000 r.p.m., for 15 min, the supernatant was collected. Protein in the supernatant was measured using a Bicinchoninic Acid Protein Assay kit (Beyotime, CA), and protein was denatured at 100 °C for 10 min with DualColor Protein Loading Buffer (Life, USA). Then, denatured protein was separated via 8–10% SDS polyacrylamide gel electrophoresis and transferred to PVDF membranes (GE Healthcare Life Sciences, UK). The membranes were blocked with TBST containing 8% non-fat milk for 2 h at room temperature (RT), incubated with specific primary antibodies at 4 °C for 16 h, and then incubated with secondary antibodies labelled with horseradish peroxidase (HRP) for 1 h at RT. Finally, an electrochemiluminescence (ECL) system (Tanon, China) was used for detection.

### CCK8 proliferation assay

Cell proliferation assays were performed using a CCK8 Cell Counting Kit (JingXin Biological Technology, Guangzhou, China) according to the manufacturer’s instructions. Briefly, cells were plated in 96-well plates at 1.0 × 10^3^ cells per well and cultured for 1–6 days. Then, 10 µl of CCK8 solution was added to each well, and cells were incubated for 3 h at 37 °C. Absorbance at 450 nm was measured with a microplate reader (SpectraMax M5 Multi-Mode Microplate Reader; Molecular Devices LLC, Sunnyvale, CA, USA). A calibration curve was prepared using data obtained from wells that contained known numbers of viable cells.

### Colony formation assays

Cells were seeded in six-well plates (500 cells per well) and cultured for 10 days. Colonies were fixed with methanol for 5 min and stained with 0.1% crystal violet for 20 min. Colonies that contained more than 50 cells were counted under a dissection microscope. The experiment was performed in triplicate for each cell line.

### Cell cycle analysis

Cell cycle stage was determined with flow cytometry using a cell cycle assay kit (Keygen Inc.). After PI staining, the cellular DNA content was determined according to the manufacturer’s instructions. The cells were distinguished as being in G0/G1, G2/M and S phases of the cell cycle based on the fluorescence intensity, and the cell cycle distribution was analysed using Cell-FIT software (BD Biosciences, San Jose, CA, USA).

### Wound healing and invasion assays

Cell migration was assessed with a scratch wound healing assay. Cells were cultured in a six-well plate. After the cells reached sub-confluence, a scratch wound was generated with a sterile micropipette tip, and the culture medium was replaced with serum-free RPMI-1640. The spread of wound closure was observed every 12 h and photographed under a microscope. Images were later analysed by determining the distance between the cells on either side of the scratch over time, and the results are presented in the figure as percent scratch closure.

For invasion assays, cells (7.5 × 10^4^) in 100 µl of serum-free RPMI-1640 medium were seeded in a Matrigel-coated chamber (BD Biosciences) with 8-μm pores present in the insert of a 24-well culture plate. FBS was added to the lower chamber as a chemo-attractant. After 18 h, the non-invading cells were gently removed with a cotton swab. Invasive cells located on the lower side of the chamber were fixed with 100% methanol for 10 min and stained with crystal violet (Weijia Biology Science and Technology Co, Ltd) for 30 min at room temperature. Five random fields per well were observed, and cells were counted under a microscope. Experiments were performed in triplicate.

### Sphere culture and sphere formation assays

Parental cells were trypsinized, washed with phosphate-buffered saline (PBS), and seeded at clonal density (1 × 10^3^ per mL) in cancer stem cell (CSC) media consisting of Dulbecco’s modified Eagle’s medium with nutrient mixture F-12 (DMEM/F12, Invitrogen, USA) supplemented with B27 (Invitrogen, USA), 20 ng/ml epidermal growth factor (Invitrogen, USA), 20 ng/ml basic fibroblast growth factor (Invitrogen, USA), and 2 mM l-glutamine (Invitrogen, USA) on ultra-low attachment plates (Sigma, USA). Conditioned medium (CM) was changed every 3–4 days, and DMSO, MK2206 or Rapamycin were added to the cultures every 48 h. The spheres were typically counted after 14 days. Only spheres >75 μm in diameter were included, if not otherwise stated.

### Animal study

Six-week-old male athymic nude mice were injected subcutaneously with 6 × 10^6^ S18-EpCAM or S18-control cells separately. The resulting tumours were examined every 3 days. After 30 days, the mice were killed and the subcutaneous tumours were resected. Tumour size was measured using calipers, and tumour volumes were calculated (*V* = 0.5 × L × W^2^). Then, the subcutaneous tumours were fixed in 10% formalin and embedded in paraffin blocks for immunohistochemistry to confirm the EpCAM protein levels.

For the in vivo metastasis assays, fourteen 3- to 4-week-old male BALB/c-nu mice were randomised into 2 groups of 7 mice each. Then, 100 μl of cell suspension containing 1 × 10^6^ S18-EpCAM or S18-NC cells was injected intravenously through the tail vein into each mouse. The experiment was terminated after 8 weeks, the mice were examined, and the liver and the lungs were removed and fixed with 10% formalin. Subsequently, consecutive tissue sections were made for each block of the liver and the lung. The sections were stained with H&E, and EpCAM expression in tumour metastasis tissues was identified using IHC. Finally, the metastatic nodules were carefully examined and counted under a microscope, and the average number of metastatic foci in each slide was calculated for each mouse and compared between S-18-NC and S-18-EpCAM groups.

Animals were housed under standard conditions and cared for according to the institutional guidelines for animal care. The experiments were performed in accordance with the guidelines of the laboratory animal ethics committee of Sun Yat-sen University.

### Statistical analysis

A *χ*^2^-test was used for correlation analysis between EpCAM expression and clinicopathological features of patients with NPC. Survival curves were plotted using the Kaplan–Meier method and compared using a log-rank test. Student’s *t*-test was used to test differences between two groups. SPSS version 17.0 software (SPSS, Inc., Chicago, IL, USA) was used for statistical analyses. *P* < 0.05 was considered to indicate a statistically significant difference (*P < 0.05; **P < 0.01; ***P < 0.001).

## Electronic supplementary material

Supplementary table 1

Supplementary figure 1

Supplementary figure 2

Supplementary Figure Legends

## References

[1] Cao SM, Simons MJ, Qian CN (2011). The prevalence and prevention of nasopharyngeal carcinoma in China. Chin. J. Cancer.

[2] Zhang LF (2015). Incidence trend of nasopharyngeal carcinoma from 1987 to 2011 in Sihui County, Guangdong Province, South China: an age-period-cohort analysis. Chin. J. Cancer.

[3] Sun X (2014). Long-term outcomes of intensity-modulated radiotherapy for 868 patients with nasopharyngeal carcinoma: an analysis of survival and treatment toxicities. Radiother. Oncol..

[4] Jia WH (2006). Trends in incidence and mortality of nasopharyngeal carcinoma over a 20-25 year period (1978/1983-2002) in Sihui and Cangwu counties in southern China. BMC Cancer.

[5] Colaco RJ (2013). Nasopharyngeal carcinoma: a retrospective review of demographics, treatment and patient outcome in a single centre. Clin. Oncol..

[6] Kohler G, Milstein C (1975). Continuous cultures of fused cells secreting antibody of predefined specificity. Nature.

[7] Herlyn M, Steplewski Z, Herlyn D, Koprowski H (1979). Colorectal carcinoma-specific antigen: detection by means of monoclonal antibodies. Proc. Natl Acad. Sci. USA.

[8] Baeuerle PA, Gires O (2007). EpCAM (CD326) finding its role in cancer. Br. J. Cancer.

[9] Trzpis M, McLaughlin PM, de Leij LM, Harmsen MC (2007). Epithelial cell adhesion molecule: more than a carcinoma marker and adhesion molecule. Am. J. Pathol..

[10] Yanamoto S (2007). Clinicopathologic significance of EpCAM expression in squamous cell carcinoma of the tongue and its possibility as a potential target for tongue cancer gene therapy. Oral Oncol..

[11] Ensinger C, Kremser R, Prommegger R, Spizzo G, Schmid KW (2006). EpCAM overexpression in thyroid carcinomas: a histopathological study of 121 cases. J. Immunother..

[12] Benko G, Spajic B, Kruslin B, Tomas D (2013). Impact of the EpCAM expression on biochemical recurrence-free survival in clinically localized prostate cancer. Urol. Oncol..

[13] Ni J (2013). Epithelial cell adhesion molecule (EpCAM) is associated with prostate cancer metastasis and chemo/radioresistance via the PI3K/Akt/mTOR signaling pathway. Int. J. Biochem. Cell Biol..

[14] Massoner P (2014). EpCAM is overexpressed in local and metastatic prostate cancer, suppressed by chemotherapy and modulated by MET-associated miRNA-200c/205. Br. J. Cancer.

[15] Matsuda T (2014). EpCAM, a potential therapeutic target for esophageal squamous cell carcinoma. Ann. Surg. Oncol..

[16] Li Y, Farmer RW, Yang Y, Martin RC (2016). Epithelial cell adhesion molecule in human hepatocellular carcinoma cell lines: a target of chemoresistence. BMC Cancer.

[17] Sun YF (2013). Circulating stem cell-like epithelial cell adhesion molecule-positive tumor cells indicate poor prognosis of hepatocellular carcinoma after curative resection. Hepatology.

[18] Went P (2006). Frequent high-level expression of the immunotherapeutic target Ep-CAM in colon, stomach, prostate and lung cancers. Br. J. Cancer.

[19] Hiraga T, Ito S, Nakamura H (2016). EpCAM expression in breast cancer cells is associated with enhanced bone metastasis formation. Int. J. Cancer.

[20] Gao J (2015). By inhibiting Ras/Raf/ERK and MMP-9, knockdown of EpCAM inhibits breast cancer cell growth and metastasis. Oncotarget.

[21] Spizzo G (2006). Overexpression of epithelial cell adhesion molecule (Ep-CAM) is an independent prognostic marker for reduced survival of patients with epithelial ovarian cancer. Gynecol. Oncol..

[22] Meng Y (2015). Cytoplasmic EpCAM over-expression is associated with favorable clinical outcomes in pancreatic cancer patients with Hepatitis B virus negative infection. Int. J. Clin. Exp. Med..

[23] Varga M (2004). Overexpression of epithelial cell adhesion molecule antigen in gallbladder carcinoma is an independent marker for poor survival. Clin. Cancer Res..

[24] Zhou N (2015). MTA1-upregulated EpCAM is associated with metastatic behaviors and poor prognosis in lung cancer. J. Exp. Clin. Cancer Res..

[25] Warneke VS (2013). Members of the EpCAM signalling pathway are expressed in gastric cancer tissue and are correlated with patient prognosis. Br. J. Cancer.

[26] Eichelberg C (2013). Epithelial cell adhesion molecule is an independent prognostic marker in clear cell renal carcinoma. Int. J. Cancer.

[27] Patriarca C, Macchi RM, Marschner AK, Mellstedt H (2012). Epithelial cell adhesion molecule expression (CD326) in cancer: a short review. Cancer Treat. Rev..

[28] Hwang EY (2009). Decreased expression of Ep-CAM protein is significantly associated with the progression and prognosis of oral squamous cell carcinomas in Taiwan. J. Oral Pathol. Med..

[29] Kimura H (2007). Prognostic significance of EpCAM expression in human esophageal cancer. Int. J. Oncol..

[30] Seligson DB (2004). Epithelial cell adhesion molecule (KSA) expression: pathobiology and its role as an independent predictor of survival in renal cell carcinoma. Clin. Cancer Res..

[31] Songun I (2005). Loss of Ep-CAM (CO17-1A) expression predicts survival in patients with gastric cancer. Br. J. Cancer.

[32] Munz M (2004). The carcinoma-associated antigen EpCAM upregulates c-myc and induces cell proliferation. Oncogene.

[33] Chaves-Perez A (2013). EpCAM regulates cell cycle progression via control of cyclin D1 expression. Oncogene.

[34] Litvinov SV (1997). Epithelial cell adhesion molecule (Ep-CAM) modulates cell-cell interactions mediated by classic cadherins. J. Cell Biol..

[35] Imrich S, Hachmeister M, Gires O (2012). EpCAM and its potential role in tumor-initiating cells. Cell Adhesion Migr..

[36] Liu Y (2012). Activation of AKT is associated with metastasis of nasopharyngeal carcinoma. Tumour Biol..

[37] Wang W (2014). Activation of Akt/mTOR pathway is associated with poor prognosis of nasopharyngeal carcinoma. PLoS ONE.

[38] Song LB (2009). The polycomb group protein Bmi-1 represses the tumor suppressor PTEN and induces epithelial-mesenchymal transition in human nasopharyngeal epithelial cells. J. Clin. Invest..

[39] Zhang LY (2013). MicroRNA-144 promotes cell proliferation, migration and invasion in nasopharyngeal carcinoma through repression of PTEN. Carcinogenesis.

[40] Zhou XM (2016). Upregulated TRIM29 promotes proliferation and metastasis of nasopharyngeal carcinoma via PTEN/AKT/mTOR signal pathway. Oncotarget.

[41] Su R (2016). Associations of components of PTEN/AKT/mTOR pathway with cancer stem cell markers and prognostic value of these biomarkers in hepatocellular carcinoma. Hepatol. Res..

[42] Fenouille N (2012). The epithelial-mesenchymal transition (EMT) regulatory factor SLUG (SNAI2) is a downstream target of SPARC and AKT in promoting melanoma cell invasion. PLoS ONE.

[43] Maetzel D (2009). Nuclear signalling by tumour-associated antigen EpCAM. Nat. Cell Biol..

[44] Yamashita T, Budhu A, Forgues M, Wang XW (2007). Activation of hepatic stem cell marker EpCAM by Wnt-beta-catenin signaling in hepatocellular carcinoma. Cancer Res..

