# Supplementary material for: Epithelial cell adhesion molecule overexpression regulates epithelial-mesenchymal transition, stemness and metastasis of nasopharyngeal carcinoma cells via the PTEN/AKT/mTOR pathway
Source: Cell Death Dis. 2018 Jan 5;9(1):2. doi: 10.1038/s41419-017-0013-8 (PMC5849035; doi:10.1038/s41419-017-0013-8)
Supplement: Supplementary file 1 — Supplementary table 1 [file 41419_2017_13_MOESM1_ESM.docx]

**Supplementary table 1. EPCAM was up-regulated in C666-1 and CNE2 cells compared with NP69 cells in RNA-Seq data.**

|  | **Mean expression** | | | **log_2_(fold change)** |  |  |
| --- | --- | --- | --- | --- | --- | --- |
| **Gene** | **NP69** | **C666-1** | **CNE2** |  | ***P*-value** | ***Q*-value** |
| EPCAM | 1.33 | 101.16 |  | 6.2469 | 0.0006 | 0.0267 |
|  | 1.14 |  | 73.27 | 6.0005 | 0.0001 | 0.0089 |
