# Supplementary figures and images for: Epithelial cell adhesion molecule overexpression regulates epithelial-mesenchymal transition, stemness and metastasis of nasopharyngeal carcinoma cells via the PTEN/AKT/mTOR pathway
Source: Cell Death Dis. 2018 Jan 5;9(1):2. doi: 10.1038/s41419-017-0013-8 (PMC5849035; doi:10.1038/s41419-017-0013-8)

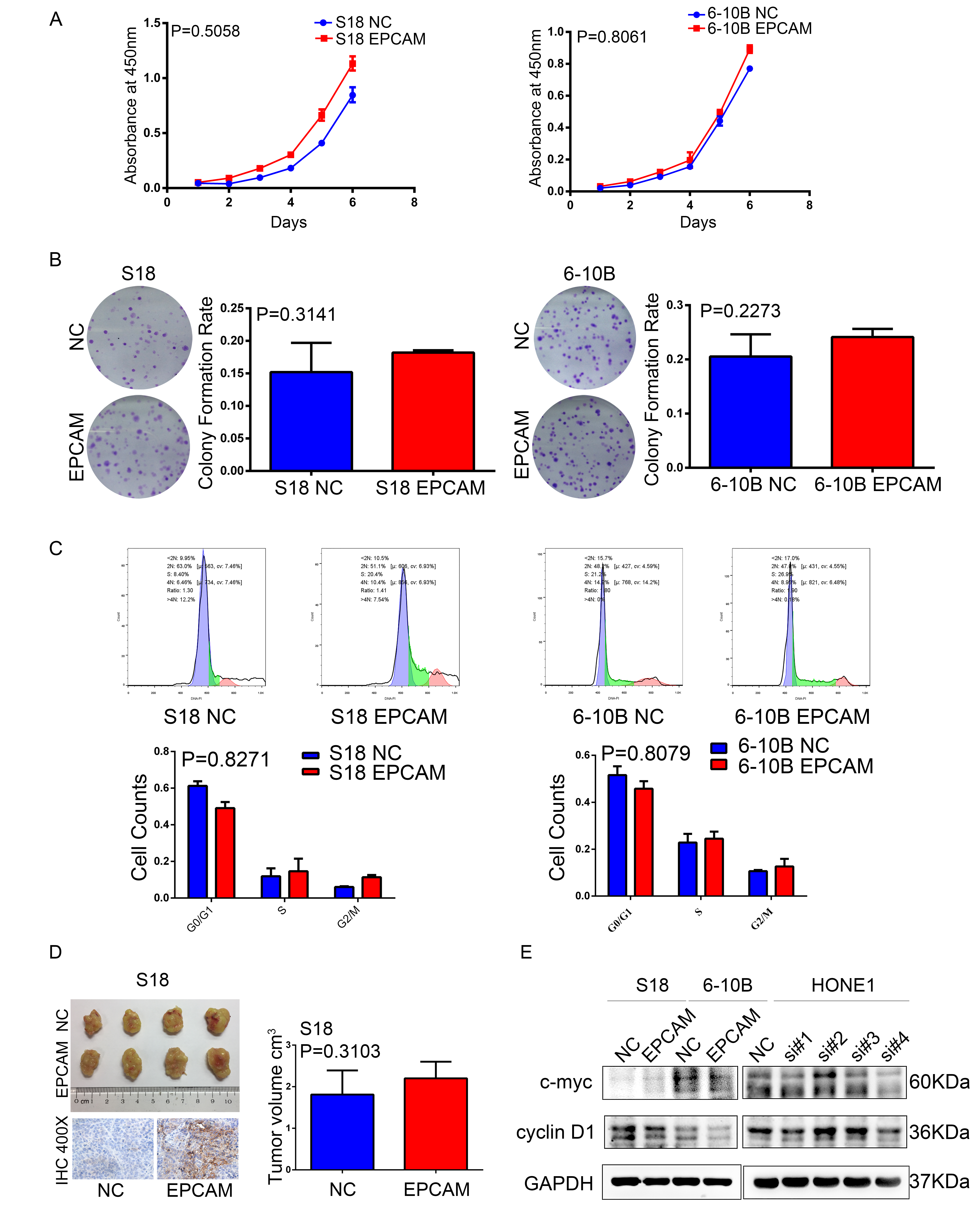

Supplement: Supplementary file 2 — Supplementary figure 1 [file 41419_2017_13_MOESM2_ESM.tif]

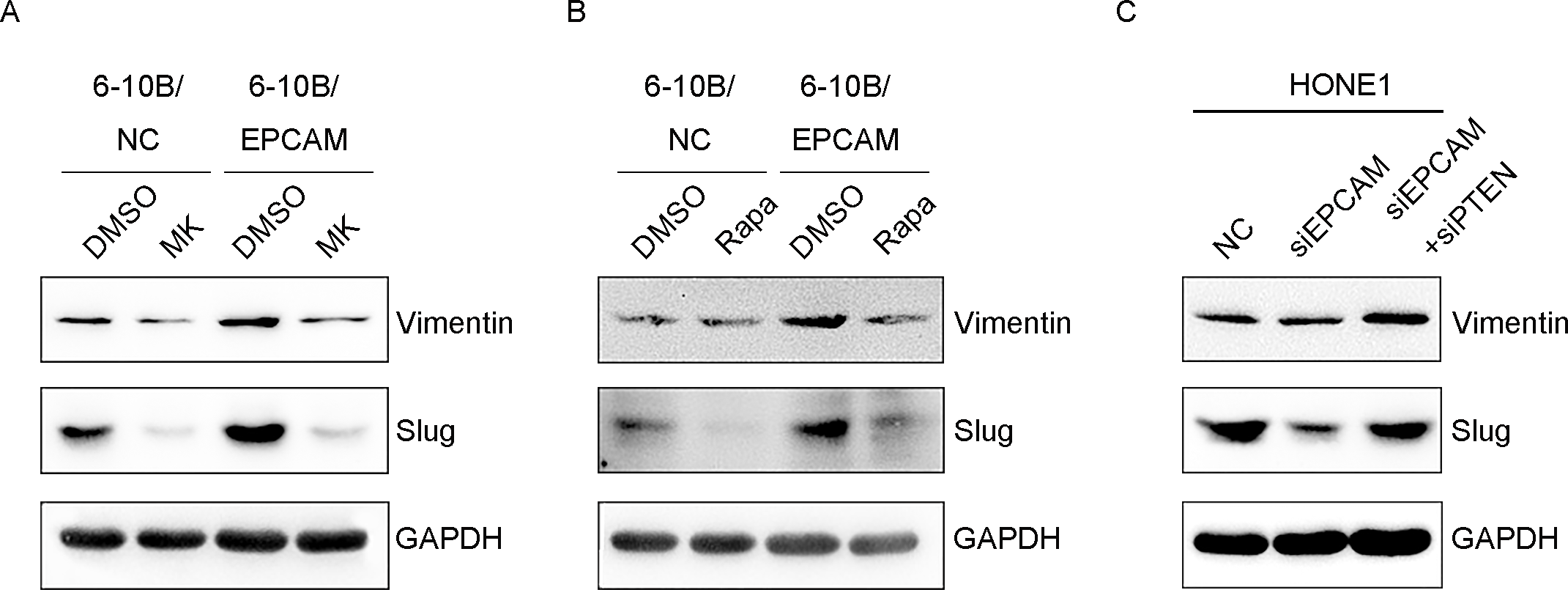

Supplement: Supplementary file 3 — Supplementary figure 2 [file 41419_2017_13_MOESM3_ESM.tif]
