# Supplementary material for: Epithelial cell adhesion molecule overexpression regulates epithelial-mesenchymal transition, stemness and metastasis of nasopharyngeal carcinoma cells via the PTEN/AKT/mTOR pathway
Source: Cell Death Dis. 2018 Jan 5;9(1):2. doi: 10.1038/s41419-017-0013-8 (PMC5849035; doi:10.1038/s41419-017-0013-8)
Supplement: Supplementary file 4 — Supplementary Figure Legends [file 41419_2017_13_MOESM4_ESM.docx]

**Supplementary figure Legends**

**Supplementary figure 1** EpCAM has no effect on the proliferation of NPC cells. (**a**) Cell viability was determined with a CCK-8 (Cell Counting Kit-8) assay. The results show that EpCAM overexpression in NPC cells has no effect on the growth of S-18 and 6-10B cells. (**b**) Assessment of the clonogenic potential of EpCAM-overexpressing cells was performed by counting colony numbers. EpCAM overexpression did not affect cell proliferation in S-18-EpCAM and 6-10B-EpCAM cells. Columns represent the mean value of 3 duplicates; bars represent standard deviation. (**c**) Flow cytometry analysis of the cell cycle distribution revealed that no significant differences were present between EpCAM-overexpressing cells and the control cells. (**d**) The role of EpCAM in NPC cell growth *in vivo* was tested in a xenograft model. Subcutaneous tumour formation was observed in 4/4 mice in the S-18-EpCAM group and 4/4 in the control group, while no significant difference in tumour number or size was found between the EpCAM-overexpressing and control groups. (**e**) Western blot analysis showed that the expression levels of c-myc and cyclin D1 were not significantly different in EpCAM-overexpressing or knockdown NPC cells compared to control cells.

**Supplementary figure 2** Inhibitors of the PTEN/AKT/mTOR pathway abolished the effect of EpCAM on NPC cell EMT. The expression levels of EMT markers Vimentin and Slug in 6-10B cells expressing EpCAM or the empty vector were assessed after pretreatment with or without 5 μM MK2206 (AKT inhibitor) (A) or 100 nM rapamycin (B). (C) Suppression of PTEN expression by siRNA restored the reduced Vimentin and Slug expression in EpCAM-depleted HONE1 cells.
